# Prevention of mother-to-child transmission of HIV infection: Views and perceptions about swallowing nevirapine in rural Lilongwe, Malawi

**DOI:** 10.1186/1471-2458-10-354

**Published:** 2010-06-21

**Authors:** Deirdre A O'Gorman, Lot J Nyirenda, Sally J Theobald

**Affiliations:** 1 Liverpool School of Tropical Medicine, Pembroke Place, Liverpool, L3 5QA, UK; 2 Reach Trust, PO Box 1597, Lilongwe, Malawi

## Abstract

**Background:**

In 2006 the World Health Organization described the status of prevention of mother to child transmission (PMTCT) service implementation as unacceptable, with an urgent need for a renewed public health approach to improve access. For PMTCT to be effective it needs to be accessible, acceptable and affordable; however research in Africa into accessibility, uptake and acceptability of PMTCT services has been predominately urban based and usually focusing on women who deliver in hospitals. The importance of involving other community members to strengthen both PMTCT uptake and adherence, and to support women emotionally, has been advocated. Urban men's and rural traditional birth attendants' (TBAs) involvement have improved uptake of HIV testing and of nevirapine.

**Methods:**

A qualitative study was carried out in a rural district of Malawi's central region to explore the views about and perceptions of PMTCT antiretroviral treatment. Semi-structured interviews and focus group discussions were held with antenatal and postnatal women, fathers, grandmothers, TBAs, community leaders and PMTCT health workers.

**Results:**

Two broad themes of findings emerged: those that relate to the hospital PMTCT service, and those that relate to the community. Trust in the hospital was strong, but distance, transport costs and perceived harsh, threatening health worker attitudes were barriers to access. Grandmothers were perceived to have influence on the management of labour, unlike fathers, but both were suggested as key people to ensure that babies are brought to the hospital for nevirapine syrup. TBAs were seen as powerful, local, and important community members, but some as uneducated.

**Conclusion:**

PMTCT was seen as a community issue in which more than the mother alone can be involved. To support access to PMTCT, especially for rural women, there is need for further innovation and implementation research on involving TBAs in some aspects of PMTCT services, and in negotiating with women which community members, if any, they would like to support them in ensuring that newborn babies receive nevirapine.

## Background

In 2006 WHO (World Health Organization) described the status of PMTCT service implementation as unacceptable, with an urgent need for a renewed public health approach to improve access to HIV prevention, treatment and care services [1]. Research in rural Malawi described that PMTCT can be an empowering process, which can profoundly influence women's experience of pregnancy and child birth [2]. For PMTCT to be effective it needs to be accessible, acceptable and affordable, and a series of steps need to take place, but there are difficulties in the implementation of each step [3].

**Figure 1:**
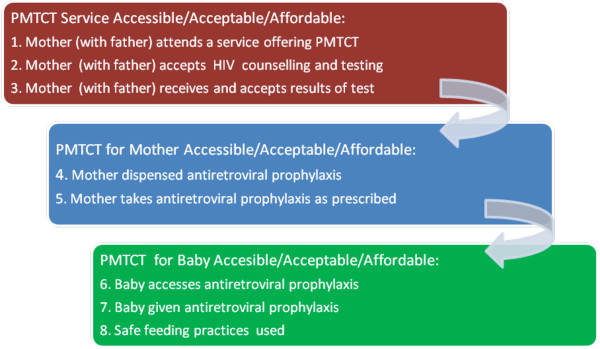
**Steps in PMTCT**.

The purpose of this qualitative study, carried out in rural Malawi, was to explore the views and perceptions of PMTCT antiretroviral treatment held by rural women, family members and health workers, in order to identify issues that facilitate and impede adherence to nevirapine. The voices and experiences of rural women have been largely neglected to date in discussions on PMTCT in Africa.

Research in Africa on the accessibility and uptake of PMTCT and strategies to improve these has focussed mainly on women who deliver in hospitals [4,3,5]. Research into adherence to PMTCT has also been predominantly urban based. Albrecht et al. [6] showed excellent adherence rates to nevirapine of 94% for mothers and 91% for babies in Lusaka, Zambia; maternal non-adherence was associated with home births, no high school education and low newborn birth weight. Among those who had home births, lack of HIV status disclosure to one's partner was a significant predictor of non-adherence.

The acceptability of PMTCT in urban African contexts, e.g. Akwa, Gabarone, Abidjan and Lusaka, has been impeded by disbelief in its effectiveness, negative attitudes of health workers and lack of male support; economic affordability constrained by distance and transport costs; and social affordability impeded by stigma, discrimination and the fear of abuse and divorce after partner disclosure [7-10]. The importance of involving other community members to strengthen both PMTCT uptake and adherence, and to support women emotionally, was advocated in urban Swaziland [11] and in the Eastern Cape, South Africa [12]. Encouraging the involvement of men in PMTCT services in Nairobi through antenatal couple counselling led to increased uptake of interventions, including the use of nevirapine by the mother [13].

Studies assessing traditional birth attendants' (TBAs) impact on PMTCT services (Fig. 1, steps 1-7) have shown increased uptake of PMTCT HIV testing and nevirapine through increasing the access of rural women in Cameroon and Tanzania to those services with a trusted member of the community. [14,15]. However, the approval of TBAs is not universal because there are issues of confidentiality, lack of acceptance of TBAs in some villages, illiteracy, training problems and uncertain impact on HIV infection rates due to the limited scope of their practices, in that more women are attended by a relative than by a TBA during labour [16,17]. In 2004 in Malawi approximately 31% of women delivered their babies at home; overall 12% of all women delivered under the care of a traditional birth attendant [18]. PMTCT services are provided through hospitals; in most places single dose nevirapine is given to antenatal mothers and they are advised to bring their newborn babies to the hospital for nevirapine syrup within 3 days of birth. In the rural Lilongwe District, where this study took place, the community practises matrilineal culture: men move to their wife's village on marriage, and the mother's brother, instead of the husband, is deemed to be in charge of their children. Traditionally, Malawian men are not encouraged to be involved in issues of pregnancy and childbirth. The overall prevalence of HIV infection is 12%, the rate in antenatal women in 2005 was 16.9%; the prevalence was lower in rural than in urban or semi-urban areas and highest in the 25-29 year old age group [19]

## Methods

Qualitative methods were used as they are well placed to identify and address issues because they are capable of exploring different views and identifying concepts and interpretations to understand the social world through people's experiences and stories [20,21]. Semi-structured interviews were used as they provide a description of the actions, ideas and experiences of the participants in their own words [22]. Focus group discussions (FGDs) were appropriate as they are helpful in finding out how and why people behave as they do [23]. The research took place in April/May 2008 in a district hospital and villages of that district, in a rural part of Malawi's central region.

Sampling was purposive and sometimes opportunistic: participants were those community members who were identified in this and other studies as influencing the actions of women in labour, i.e. antenatal and postnatal women, men who are fathers, traditional birth attendants (TBAs), grandmothers, community leaders and PMTCT health workers. TBAs were identified by hospital staff; mothers, fathers, community leaders and grandmothers were indentified in the TBAs' and surrounding villages. Each TBA had an antenatal clinic with a delivery room, and they advised which day to come to see the antenatal women attending their clinics. A group of fathers playing a traditional game in one village agreed to participate in a focus group. TBAs and chiefs identified older women in their communities. Interviews and focus group discussions (FGDs) took place in the hospital (Table 1), in TBAs' clinics, under trees, in an empty school and in the research vehicle (in all cases privacy and confidentiality were assured).

**Table 1:** Participants: FGDs/interviews

70 Participants:	Focus Group Discussions	Interviews
		4 antenatal women
Women	29 antenatal women in 3 FGDs	5 grandmothers
	6 postnatal women in 1 FGD	4 PMTCT health workers
		5 traditional birth attendants
	(total 35)	(total 18)

		3 fathers
Men	9 fathers in 1 FGD	3 church leaders
		2 chiefs
	(total 9)	(total 8)

Written consent was obtained from every participant in their local language; participation was voluntary and under an agreement to keep details confidential. 3 Interviews in English were conducted by D.A.O'G., trained in qualitative research; the remaining interviews and all the FGDs were conducted by two trained, experienced Malawian research assistants in Chichewa, the local language, and all were recorded. Data was transcribed directly into English by the research assistants: translations were periodically checked through discussion and by comparing transcripts to the notes taken (in English) at the time of the interview/FGD. MAXQDA software was used to facilitate coding and mapping of data. The framework analysis [24] was used in order to ensure comprehensive, rigorous, logical analysis of the data.

Ethical approval for the research was obtained from the ethics committee of Liverpool School of Tropical Medicine in England and from the National Health Sciences Research Committee, Malawi.

## Results

2 broad themes of findings emerged: facilitators and barriers that relate to the hospital PMTCT service, and those that relate to the community. All participants, with the exception of 2 grandmothers, knew that there is a medicine (nevirapine) for pregnant women to take to prevent HIV from passing into their baby and a medicine for the baby.

### Factors affecting hospital attendance

Trust in the hospital and its advice was expressed by 14 of the 22 interviewees, and in all FGDs:

*'I trust them because they are educated and they know what and how to do whenever you come up with a problem.' *(Antenatal woman, FGD)

Distance from the hospital and transport costs constitute barriers to access. The villages where the data collection took place were between 5 and 15 kilometres from the hospital: this proved to be a physical barrier to delivering in the hospital, particularly if labour starts at night, and to bringing a baby there for nevirapine syrup after birth. An issue particularly affecting the baby's chance of getting nevirapine at the hospital was ill-health of the mother after delivery, for example inability to walk because of pain or bleeding. The attitude of health workers in the hospital was also an issue for both men and women. Health workers were described as harsh and threatening, although one antenatal woman said her experience there was a welcoming one. Others described how the health workers did not seem to respect them and could ill-treat them:

'[The health workers] *shout, insult women even when the time has come for the women to deliver. They even leave you to deliver on your own while they are shouting bad words at you. There are times when you try to call them for help, they do not come' *(Postnatal woman, FGD)

*'...it can be when the mother delivered at home, she can have fear to go to the hospital *[for nevirapine syrup], *because most of us fear to be shouted at by doctors because of delivering at home' *(Antenatal woman, interview)

### Community Factors

#### Fathers/men

Fathers are often present when women start labour however they were not seen to have much power over what women do in pregnancy or in labour: they hand that responsibility over to their mother or mother-in-law and other women in the village, including the TBA. The lack of male involvement in delivery was a cross cutting theme in the qualitative analysis. Illustrative quotes include:

*'I never had power to decide where to go with my wife to have her baby it's the responsibility of my mother in law.' *(Man, interview)

*'Women ... are there together with the TBA according to their tradition. We men are not allowed to be present' *(Chief, interview)

Fear of divorce was often mentioned by both men and women as a reason for women not to disclose their HIV test result and their and their baby's need for nevirapine: '...*sometimes it is because she fears that her husband will realise that she is positive and he... will divorce her when he is a person without love*' (Postnatal woman, FGD)

#### Grandmothers

All participants said that grandmothers play a central role in delivery of babies at home or with the TBA. The pregnant woman's mother or mother in law is called at the start of labour; either of those may call on the assistance of other older women in the village for the delivery, or may escort the woman to the TBA's clinic and these women are involved in any decision about the progression of the labour. Grandmothers differed in their attitude to PMTCT: one grandmother said that some of them only believe what they heard from their parents; another acknowledged that times are changing requiring new attitudes and actions:

*'Old things were good but these days are different. To say no to swallowing, it's not wisdom.' *(Grandmother, interview)

Participants were asked who should know that the baby needs to be brought to the hospital: only 2 women said that they alone should know; otherwise women, men, PMTCT health workers and TBAs said both parents, or parents and a grandmother, or parents and an aunt and other relatives, or parents and the TBA should know. Likewise when asked who should bring the baby to the hospital for nevirapine syrup this was seen as the sole responsibility of the mother by only one woman; instead frequently it was seen as both the mother's and father's task, or the task of the grandmother or other relatives. Two TBAs suggested they could do it. Only one man suggested that men cannot bring a baby to the hospital.

#### Stigma

The influence of their peers on women can be strong. Being known to be HIV infected and fear of stigma can impact on a woman's decision on whether or not to take nevirapine. Illustrative quotations include:

*'The woman cannot swallow because of fear of stigma hence she doesn't disclose her cell status to the family members' *(Antenatal woman, FGD)

'...*some do not swallow because of the following reasons: forgetfulness and fear of stigma from the general community' *(PMTCT worker)

*'...the community can be suspecting me that I am positive and they may be laughing at me' *(Antenatal woman, interview)

#### Traditional Birth Attendants

Women said they choose to deliver at the TBA's clinic as it is nearby and because they are not shouted at by the TBA; also because of belief that the TBA's care is as good as or better than care at the hospital, and because some women perceived TBAs to be more experienced than the hospital workers. TBAs are sometimes credited with powers that hospitals are not, e.g. the ability to counteract a curse, or to be able to change the position of an unborn baby who is not lying in a position for easy delivery:

*'There are times when within a family, people hating each other to the point that they use magic to kill the unborn baby. The TBA reverses the situation using herbs where ...the hospital has failed to recognise the problem.' *(Postnatal woman, FGD)

Delivering at the TBA's clinic can be a way for women to avoid adhering to PMTCT instructions to deliver at the hospital:

*'you know most people believe that those with HIV are prostitutes and that mentality *(is) *established in many people here: once one is tested positive at the ANC they stop going there instead they come at the TBA' *(Antenatal woman, interview)

Lack of adherence to nevirapine is also blamed on the lack of supply at the TBA's clinic:

*'It is because the TBA does not provide them so the woman can't swallow it.' *(Antenatal woman, FGD)

TBAs are important members of the community; one man described their role by saying '*TBAs are part of us here*'; a man in a FGD said that they *'are our mothers'*, however, another man said they are '*after money'*. Almost all participants thought that TBAs should play some role in PMTCT, from their recently established role of referring antenatal women for PMTCT testing and referring those who are infected to hospital for delivery, to counselling, educating, dispensing PMTCT drugs and reminding women to swallow them, and taking the baby to the hospital for nevirapine syrup, if they were trained. In one FGD some women suggested that TBAs should be the people who do testing and who dispense nevirapine, but this was rejected by others in the FGD as TBAs do not have the equipment to test for HIV. Two PMTCT health workers expressed that the advantages of involving TBAs were that they live and work in the community and could provide this service to women who don't live near a hospital; the other two PMTCT health workers were less enthusiastic about the idea because of TBAs' lack of education and lack of knowledge about PMTCT.

*'I think I would prefer the TBAs to be involved fully in this programme because they are the ones who lives with the community, they are the ones who do the deliveries in the community' *(PMTCT worker)

TBAs themselves were keen to be involved; they acknowledged their lack of equipment for HIV testing, but were eager to help antenatal women by counselling and providing nevirapine.

## Discussion

The findings of this study show that there are many perceived barriers to swallowing nevirapine: some concerning the PMTCT service in the hospital, others community based and some relating to both the hospital service and the community. The qualitative nature of this study allowed exploration of the views and perceptions of members of the community not usually included in research about PMTCT.

A key theme of this study is that participants do not consider that PMTCT is the sole responsibility of the mother. Only one woman and one chief thought that bringing the baby to a health facility to receive nevirapine syrup was a role for the woman alone. The constraints of distance, health worker attitudes, and pain and bleeding postpartum mirror the issues found by Leigh et al [25] in their study of access to emergency obstetric care in Malawi. Most participants, including men, suggested that the husband could bring the baby, and that other family members and traditional birth attendants could also be involved. This suggestion contrasts starkly with the lack of role and responsibility for men in their wives' labour and delivery described in this and another study in Malawi [26], and appears to mark a change from the traditional attitude to men's participation. Women discussed issues around forgetting to swallow nevirapine because they had nobody to remind them: the presence of fathers when many women start labour makes them a clear choice to remind women. This willingness of men to be included presents an opportunity to engage them in the antenatal and postnatal care of women and babies as maternity services expand to include prevention of HIV infection.

Grandmothers were one of the strongest influences on the behaviour of women in labour who deliver at home: the success of PMTCT will be impeded if these influential people are not informed about PMTCT and of its importance [11,12]. They are often the first people to be informed by a woman that she has started labour, and so, like husbands, are ideally placed to remind women to swallow nevirapine for those women who choose to disclose their status to their mother or mother in law.

The perception that PMTCT is not the sole responsibility of pregnant women is not reflected in the way the service is currently delivered through the hospital's antenatal clinic, which uses the biomedical model of a health provider with a patient who is an non-contributing, though cooperative, recipient of care [27]. The influences on a pregnant woman are strong: her peers, grandmothers, family, traditional birth attendants and culture, yet, as observed in this study, she attends the hospital for antenatal care alone. Women play important productive and reproductive roles in society [28]: the biopsychosocial model of health care in which societal values and culture are considered can offer solutions for a more appropriate PMTCT service, as pregnant women are part of a family and a community, and babies are born into that family and community [29].

Some HIV services already take place in community settings removed from health facilities. Voluntary counselling and testing sites are often geographically separate from HIV treatment services, and counsellors may not be medically trained; likewise the HIV counselling and testing step in PMTCT could be separate from the dispensing of antiretroviral medicine to the mother, and could be carried out by community members, for example, the TBAs. For those women who use them, TBAs are ideally placed to undertake the counselling and testing (CT) role: they are already involved in antenatal care, they are local, they are generally trusted, and, as has been found elsewhere, they are willing [30]. This should be no more stigmatising than current services. TBAs, however, have not received confidentiality training, which, as has been noted [16], is a necessity for a counsellor, and further research would be required to assess the impact of TBAs and barriers to their inclusion in PMTCT.

If TBAs and family members were allowed to store nevirapine syrup then more babies could access it, as many of the barriers to babies getting nevirapine in the hospital reflected distance to the hospital, fear of abuse from the PMTCT workers there and stigma arising from making that journey. This would mirror the guardian-based provision of anti-tuberculosis drugs in the DOT (directly observed treatment) programme [31], which has been successful in Malawi [32,33]. The local storage of nevirapine would also mean that nevirapine syrup is available for the baby very soon after birth: giving nevirapine to the baby soon after delivery is more effective than later [34]. In Malawi in 2004 almost three quarters of home deliveries were in rural areas and 58% of those delivering at home had received no antenatal care. TBAs, who conducted 29% of rural deliveries in 2004, live in villages and are known in communities, and are in a much better position to access pregnant women than hospital based PMTCT workers are, possibly by active case finding; likewise family members are trusted and accessible for many women. The TBAs in this study had recently received training from midwives in the district hospital about the referral of antenatal patients for PMTCT testing; further involvement and training of TBAs could be part of the task-shifting programme of WHO, already established in Malawi, and would relieve the workload of health workers that are already in short supply [35].

**Figure 2:**
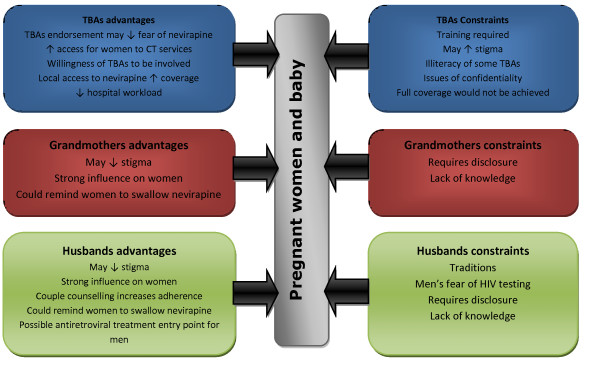
**Advantages and constraints to involving TBAs, grandmothers and fathers in PMTCT**.

The constraints to the involvement of family members and TBAs need to be considered: participation will not be applicable to all family members or all TBAs, and will need to be negotiated with women so that it reflects their individual wishes, realities and personal choices on disclosure. The possible benefits and limiting factors to their involvement in PMTCT are summarised below in Figure 2.

The Malawian PMTCT package requires that women attend formal health services before and 3 days after birth; some effort has been made to include men in the service through couple counselling. However the reality for some women is that all their maternity services are community based, through clinics run by TBAs: attending a hospital for maternity services is not a feasible choice, and their decisions about maternity care are mediated through the community in which they live.

### Limitations

This is a small, qualitative study carried out over a short time in one rural district of Malawi which cannot fully elucidate all aspects of this issue. It was carried out in a rural area because rural women are least likely to adhere to nevirapine; the findings may not be applicable to urban areas where services and barriers are likely to be dissimilar, and they also may not apply to other rural areas where traditions and influences are different. Time and logistical constraints meant that data were transcribed directly into English which did not allow independent verifications of translations.

## Conclusion

This study suggests that PMTCT is a community issue which requires a community response. PMTCT services are not currently accessible, acceptable and affordable for all rural women; innovative strategies which include the involvement of different community members are needed to improve this, and therefore help this community to prevent HIV infection in its children. To meet WHO's call for stronger more public health orientated approaches to PMTCT, and to increase the practical knowledge-base on what works for poor rural women, there is an urgent need for further implementation research to evaluate and scale-up successful approaches. This research shows the importance of involving TBAs in some aspects of PMTCT services, and asking women which community members (for example grandmothers, husbands, TBAs), if any, they would like to support them in ensuring their newborn babies receive nevirapine. Without further innovation in this area PMTCT will remain inequitable and ineffective and out of reach of many poor rural women in Sub-Saharan Africa with wide reaching implications for future generations.

## Competing interests

The authors declare that they have no competing interests.

## Authors' contributions

D.A.O'G. designed and led the study, and drafted this paper. LN supported the study process, and the analysis and commented on the paper. ST supported overall study design, including analysis, and paper writing. All authors have read and approved the final manuscript.

## Appendix 1

Topics discussed in FGDs and semi-structured interviews

1. Introduction of moderator and recorder, explanation of recording equipment and issues of confidentiality.

2. Description of HIV transmission from mother to baby by moderator.

3. Knowledge about PMTCT, particularly ART in PMTCT.

Who needs it? (e.g. some or all women, some or all HIV positive women?)

Who provides it? (Is that a trustworthy source?)

Does it work? (If so, how well does it work? If not, why doesn't it work?)

4. Description of labour and delivery at home:

Who is present?

Who does what? (Who is in charge? If there's a problem, who decides what to do?)

Why at home and not in a health centre?

5. Swallowing NVP in labour:

What makes it easy/difficult? (e.g. the presence of other people - does the presence of a grandmother help/hinder?)

If difficult, what would have to be different to make it easy? (e.g. TBA involvement, partner disclosure/involvement)

6. Bringing the baby for NVP syrup to the health facility:

What makes it easy/difficult? (e.g. health facility opening hours, stigma)

Who knows it needs to be done?

Who should do it? (mother, father, other relative)

If difficult, what needs to change about it?

7. WHO's expanded PMTCT guidelines - explanation by moderator:

How will that fit in with current labour practices?

What are the issues about a newborn baby taking treatment after birth for a week or 4 weeks?

What are the issues about pregnant women taking treatment every day for the last 3 months of their pregnancies?

8. TBAs' role in PMTCT

Should they be involved?

If yes, how?

## Appendix 2

Framework analysis consists of 5 stages: [36]

1. Familiarisation with the data. 2. Identification of a thematic framework. 3. Coding. 4. Charting. 5. Mapping/Interpretation.

## Pre-publication history

The pre-publication history for this paper can be accessed here:

http://www.biomedcentral.com/1471-2458/10/354/prepub
